# Supplementary material for: Asynchronous magnetic resonance elastography: Shear wave speed reconstruction using noise correlation of incoherent waves
Source: Magn Reson Med. 2022 Oct 27;89(3):990–1001. doi: 10.1002/mrm.29502 (PMC9792433; doi:10.1002/mrm.29502)
Supplement: Supplementary file 1 — DATA S1 MATLAB code used to generate the numerical simulations of Figure 1 [file MRM-89-990-s002.zip › k-Wave/helpfiles/example_na_source_smoothing.html]

Source Smoothing Example (k-Wave)


# Source Smoothing Example

This example illustrates how spatial smoothing can be used to reduce discrete sampling artifacts.

- Open the file in the MATLAB Editor
- Run the file in MATLAB

For a more detailed discussion of this example, see Treeby, B. E. and Cox, B. T., "A k-space Green's function solution for acoustic initial value problems in homogeneous media with power law absorption," *J. Acoust. Soc. Am.*, vol. 129, no. 6, pp. 3652-3660, 2011.

## Contents

- Bandlimited interpolation
- Reducing visible oscillations via source smoothing

## Bandlimited interpolation

When an initial pressure distribution with sharp gradients is used within k-Wave, oscillations that are not intuitively expected can appear in the numerical solution for the pressure field. The occurrence of these oscillations can be understood by considering the initial pressure distribution as the discrete sampling of a truncated Fourier series with a finite number of expansion coefficients (this continuous distribution is often called the band-limited interpolant). It is this band-limited interpolant that is propagated in Fourier space, thus when the function is discretely sampled at the grid nodes, oscillations can appear in the solution. This is illustrated below where the discrete pressure is shown with a stem plot and the band-limited interpolant overlaid with a solid line.

Oscillations of this type are not unique to the solution method used in k-Wave. They are similarly encountered in other spectral methods in which the discretisation of the computational domain causes the corresponding Fourier space to become discrete and bounded (i.e., there is a finite number of spatial wavenumbers the computational grid can support).

## Reducing visible oscillations via source smoothing

The occurrence of oscillations is deterministic and intrinsic to the representation of the wavefield in a truncated Fourier basis. However, in some situations a practical method of reducing the visible oscillations may be desirable. For example, in many cases the discrete initial conditions represent an approximation to a continuous function for which the analogous pressure field does not contain any oscillations. The oscillations can be reduced by changing the characteristics of the band-limited interpolant by applying a window in the spatial frequency domain. This modifies the properties of the bounded Fourier space by forcing the expansion coefficients to decay. Selecting the most appropriate window requires a trade off between maintaining an acceptable main lobe width (which affects the amount of smoothing) whilst minimising the side lobe levels (which affects the observable oscillations). Note, the rectangular window is equivalent to the unwindowed case and produces the smallest possible main lobe width.

The figure below illustrates the initial pressure distribution, recorded time series, and the corresponding amplitude spectrum for the propagation of a spatial impulse in one dimension. The middle and lower panels show the same example with the initial pressure smoothed using frequency domain Hanning and Blackman windows, respectively. The magnitudes of the smoothed initial pressure distributions have been corrected by the corresponding coherent gain of the windows. The Blackman window has a lower side lobe level and thus the oscillations are less apparent. However, it also has a larger main lobe width and thus the spatial delta function appears more heavily smoothed. Both windows have high side-lobe roll-off rates so the oscillations decay quickly away from the peak. A large number of other variations are also possible by choosing from the families of existing window functions. For minimising oscillations, the Blackman family of windows is a good choice. This is the default window used by `smooth` which is used by default on `source.p0` within the k-Wave simulation functions.
